# The SQSTM1/p62 UBA domain regulates Ajuba localisation, degradation and NF-κB signalling function

**DOI:** 10.1371/journal.pone.0259556

**Published:** 2021-11-04

**Authors:** Melanie A. Sultana, Carmel Cluning, Wai-Sin Kwong, Nicole Polain, Nathan J. Pavlos, Thomas Ratajczak, John P. Walsh, Jiake Xu, Sarah L. Rea

**Affiliations:** 1 Neurogenetics Laboratory, Harry Perkins Institute for Medical Research, University of Western Australia, Nedlands, Australia; 2 Department of Endocrinology and Diabetes, Sir Charles Gairdner Hospital, Nedlands, Western Australia, Australia; 3 Centre for Molecular Medicine and Innovative Therapeutics, Murdoch University, Murdoch, Western Australia, Australia; 4 School of Biomedical Sciences, University of Western Australia, Crawley, Western Australia, Australia; 5 Medical School, University of Western Australia, Crawley, Western Australia, Australia; 6 School of Pathology and Laboratory Medicine, University of Western Australia, Perth, Western Australia, Australia; 7 Perron Institute for Neurological and Translational Science, Centre for Neuromuscular and Neurological Disorders, The University of Western Australia, Nedlands, Western Australia, Australia; King Faisal Specialist Hospital and Research Center, SAUDI ARABIA

## Abstract

The LIM-domain containing protein Ajuba and the scaffold protein SQSTM1/p62 regulate signalling of NF-κB, a transcription factor involved in osteoclast differentiation and survival. The ubiquitin-associated domain of SQSTM1/p62 is frequently mutated in patients with Paget’s disease of bone. Here, we report that Ajuba activates NF-κB activity in HEK293 cells, and that co-expression with SQSTM1/p62 inhibits this activation in an UBA domain-dependent manner. SQSTM1/p62 regulates proteins by targeting them to the ubiquitin-proteasome system or the autophagy-lysosome pathway. We show that Ajuba is degraded by autophagy, however co-expression with SQSTM1/p62 (wild type or UBA-deficient) protects Ajuba levels both in cells undergoing autophagy and those exposed to proteasomal stress. Additionally, in unstressed cells co-expression of SQSTM1/p62 reduces the amount of Ajuba present in the nucleus. SQSTM1/p62 with an intact ubiquitin-associated domain forms holding complexes with Ajuba that are not destined for degradation yet inhibit signalling. Thus, in situations with altered levels and localization of SQSTM1/p62 expression, such as osteoclasts in Paget’s disease of bone and various cancers, SQSTM1/p62 may compartmentalize Ajuba and thereby impact its cellular functions and disease pathogenesis. In Paget’s, ubiquitin-associated domain mutations may lead to increased or prolonged Ajuba-induced NF-κB signalling leading to increased osteoclastogenesis. In cancer, Ajuba expression promotes cell survival. The increased levels of SQSTM1/p62 observed in cancer may enhance Ajuba-mediated cancer cell survival.

## 1.0. Introduction

Mutations in *SQSTM1* affecting the region coding for the UBA domain of SQSTM1/p62 are reported in 25–50% of patients with familial Paget’s disease of bone, with the majority of mutations reported to date causing either an amino acid substitution within the UBA domain or truncation of the translated protein [1,2]. In addition to genetic factors, chronic infection with measles virus appears to be involved in pathogenesis in some cases [3]. We have shown that UBA mutant SQSTM1/p62 proteins have a decreased capacity to repress the transcriptional activity of NF-κB [4]. Specificity in signalling is partly endowed by expression of intermediate proteins known as scaffolds or adaptors [5]. SQSTM1/p62 is a scaffold protein involved in regulation of the transcription factor nuclear factor kappa B (NF-κB) in response to nerve growth factor, interleukin-1, tumour necrosis factor (TNF) α and receptor activator of NF-κB ligand (RANKL) [6], forming signalling complexes that typically contain TNF-receptor associated factor 6 (TRAF6), SQSTM1/p62 and atypical protein kinase C ζ (aPKC). More recently, the related proteins Ajuba and LIMD1 have been identified as additional components of SQSTM1/p62 protein complexes involved in signalling to NF-κB and activator protein-1, respectively. Both Ajuba and LIMD1 can interact with SQSTM1/p62 directly [7,8]. Ajuba and LIMD1 are members of the Zyxin/Ajuba family of LIM-domain containing proteins with roles in cell-cell adhesion, cell signalling and cell migration [9]. LIM proteins shuttle into the nucleus where they may influence cell lineage fate determination, however the precise nuclear and cytoplasmic roles of these proteins is not clear [10].

Ajuba forms a complex with aPKC, SQSTM1/p62 and TRAF6 in response to IL-1 stimulation and the association of Ajuba with SQSTM1/p62 was important for effective activation of aPKCζ and subsequently NF-κB [7]. Activated NF-κB is then able to translocate to the nucleus where it can affect gene transcription. The activation of NF-κB is critical for osteoclast formation. Previously, we, and others, have shown that expression of wild type SQSTM1/p62 attenuates NF-κB activity [4,11–14]. Thus, p62 has a dual role in regulating NF-κB. Negative regulation of NF-κB in osteoclasts appears to be partly mediated by SQSTM1/p62 forming a scaffold between the de-ubiquitinating enzyme CYLD and TRAF6, allowing CYLD to de-ubiqutinate TRAF6 and thereby reduce NF-κB activation [15]. In this study, we describe that SQSTM1/p62 represses Ajuba-induced NF-κB signalling in a UBA domain-dependent manner, leads to mislocalisation of Ajuba, that Ajuba is degraded by autophagy and SQSTM1/p62 protects Ajuba from degradation. We propose that SQSTM1/p62 expression recruits Ajuba into holding structures that inhibit signalling and are not destined for degradation. We discuss the implications of our finding for various disease states involving Ajuba.

## 2.0. Material and methods

### 2.1. Transfections

Unless stated otherwise, HEK293 cells were transfected in 25 cm^2^ flasks as follows. Culture medium was aspirated from cells prior to transfection and replaced with 5 mL DMEM supplemented with 10% FCS. A volume of 12 μL of Lipofectamine 2000 transfection reagent (Invitrogen) was mixed with 238 μL of OPTIMEM (Invitrogen) and the solution incubated at room temperature for 5 min. Four to five μg of plasmid DNA was added to OPTIMEM to a final volume of 250 μL. The DNA solution was then added to the Lipofectamine 2000 solution and the components were allowed to form complexes for 20 min at room temperature prior to addition to the cells and incubation for 4–6 h at 37°C, with 5% CO_2_. Transfection medium was then aspirated and fresh DMEM containing 10% FCS and antibiotics was applied to the flasks. Prior to sampling, cells were incubated at 37°C with 5% CO_2_ for a minimum of 24 h post transfection.

### 2.2. Luciferase reporter assays

Briefly, HEK293 cells were seeded at a density of 4 x 10^4^ per well in 96-well plates. The following day cells were transfected with 30 ng each of FLAG-TRAF6 and His-Ajuba DNA and 30 ng of either pcDNA3.1, or pcDNA3.1-FLAG-p62 (wild type or ΔUBA) plus 10 ng of p3κB-luciferase or pAP1-luciferase and 2.5 ng pRenilla-luciferase. Forty-eight hours post-transfection, cells were processed for dual-luciferase readings using the Dual-Glo luciferase assay kit according to the manufacturer’s protocol (Promega, USA). Data presented is the mean +/- SEM of 3 independent experiments performed in quadruplicate.

### 2.3. Co-immunoprecipitations

Unless otherwise stated, HEK293 cells were transfected with equal amounts of expression plasmids for FLAG-SQSTM1/p62 and HIS-Ajuba. For experiments with varying amounts of SQSTM1/p62 expression plasmids, total DNA transfected was kept constant with the addition of pcDNA3.1. Forty-eight hours post transfection, media was aspirated and cells were washed twice with ice-cold PBS. Cells were then lysed in 1 mL of RIPA buffer [150 mM Trs-HCl pH7.5, 150 mM NaCl, 0.1% SDS, 0.5 mM EDTA, 0.5% sodium deoxycholate, 1% Triton-X] with added proteinase inhibitors and passed ten times through a 25-gauge needle, cell lysates were then cleared by centrifugation for 30 mins at 14,000 rpm at 4°C. Clarified lysates were rotated with 25 μL washed Sepharose G beads for 2 h at 4°C and centrifuged for 1 min at 1000 rpm at 4°C. The protein content of lysates was determined and between 750 μg and 1 mg of protein was incubated overnight with antibody [2 μL mouse anti-FLAG or anti-Histidine (Sigma Aldrich)] at 4°C with rotation. The following day lysates were incubated with 25 μL washed Sepharose beads for 2 h at 4°C with rotation. Beads with antibody-captured proteins were pelleted and washed 3 times with RIPA buffer prior to protein elution into SDS sample buffer. Eluted proteins and loading controls (input, 20 μg) were separated by electrophoresis through 10% SDS polyacrylamide gels and then transferred to a nitrocellulose membrane at 4°C overnight.

### 2.4. Ajuba degradation experiments

For experiments investigating dose-dependent effects of SQSTM1/p62 expression on Ajuba levels HEK293 cells were transfected with equal amounts of pcDNA4/Xpress-HIS Ajuba with increasing amounts of an expression plasmid for either wild type SQSTM1/p62 (1 μg; or 4 μg) or UBA-deficient (ΔUBA) SQSTM1/p62 (1 μg or 4 μg) or empty vector (EV 4 μg). Thirty hours post-transfection cells were lysed in RIPA buffer and western blot analyses were performed using anti-HIS (Ajuba), anti-FLAG (SQSTM1/p62) and anti-α-tubulin antibodies. For experiments investigating Ajuba degradation via autophagy HEK293 cells were transfected with equal amounts of pcDNA4/Xpress-HIS Ajuba and wild type SQSTM1/p62, ΔUBA SQSTM1/p62 or empty vector (EV). Twenty-four hours post-transfection cells treated with 10 μM MG132 or serum starved overnight or left untreated. Cells were lysed in RIPA buffer and western blot analyses were performed using anti-HIS (Ajuba), anti-FLAG (SQSTM1/p62) and anti-α-tubulin antibodies.

### 2.5. Subcellular fractionation assays

HEK293 cells were transfected with equal amounts of expression plasmids for FLAG-SQSTM1/p62 and HIS-Ajuba. Forty-eight hours post transfection, media was aspirated and cells were washed twice with ice-cold PBS. Cells were fractionated using a Subcellular fractionation kit for cultured cells according to manufacturer’s instructions (Thermo Scientific). Equal amounts of each fraction were separated through 10% SDS polyacrylamide gels and then transferred overnight at 4°C to nitrocellulose membranes.

### 2.6. Western blot analysis

Antibodies to α-tubulin, FLAG and HIS_6_ were purchased from Sigma-Aldrich, and anti-Ajuba antibody was purchased from Cell Signalling. For all western blots, membranes were incubated in blocking solution [5% (w/v) skim milk powder (SMP) in TBS (10 mM Tris, pH 7.5, 150 mM NaCl)] for 1 h, followed by incubation for 1 h with primary antibody at room temperature. Primary antibodies were diluted 1: 10,000 (anti-HIS and anti-α-tubulin), 1:5000 (anti-Ajuba) in 5% SMP in TBS-T (TBS with 0.05% Tween 20). or 1:10,000 (anti-FLAG) in 3% SMP in TBS. Membranes were washed 3 times in TBS-T and then incubated with appropriate horseradish peroxidase (HRP)-conjugated secondary antibodies (Sigma Aldrich) in 3% SMP in TBS-T. Membranes were washed three times with TBS-T prior to development using the Western Lightning Chemiluminescence reagent plus (PerkinElmer Life Sciences).

### 2.7. Statistical analyses

Post-hoc ANOVA statistical analyses were performed using IBM SPSS (Version 24) with significance set to p < 0.05.

## 3.0. Results

### 3.1. Ajuba activation of NF-κB signalling is abrogated by SQSTM1/p62 expression

**Fig 1:**
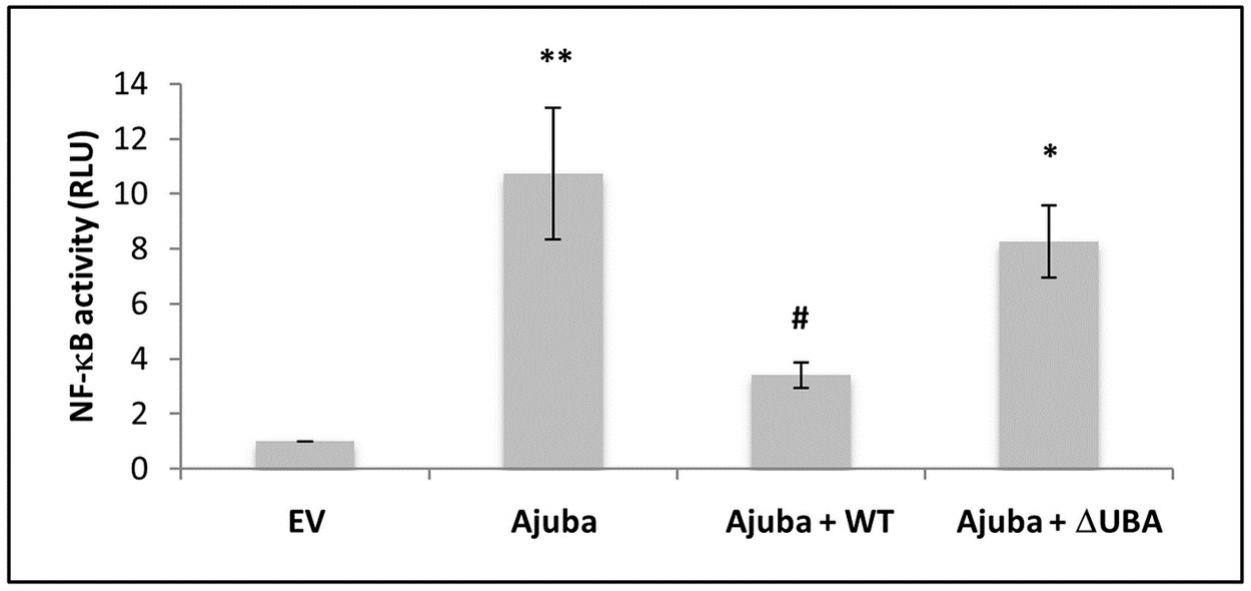
Ajuba activation of NF-kB is attenuated by co-expression with p62. HEK293 cells were transfected with expression plasmids for TRAF6 and Ajuba, with empty vector (EV), WT p62 or DUBA p62, along with Renilla-luciferase and an NF-kB firefly luciferase reporter. Firefly readings were normalised to renilla and then EV (set as 1.0). Data are the mean +/- SEM of 3 independent experiments performed in quadruplicate. *p < 0.04 and **p < 0.001 compared with EV and # p <0.04 compared with Ajuba.

Ajuba is an important component of the aPKC, TRAF6, SQSTM1/p62 complex involved in NF-κB signalling [7]. To determine whether Ajuba, affects TRAF6-induced NF-κB signalling, we co-transfected Ajuba, TRAF6 and p62 (wild type or UBA deficient) or pcDNA3.1 empty vector. By co-transfecting p62, TRAF6 and Ajuba we aimed to express roughly equivalent quantities of each protein to allow these naturally occurring signalling complexes to occur. When compared with empty vector transfected cells not expressing Ajuba, cells expressing Ajuba had significantly higher NF-κB activation (Fig 1A). Co-expression of wild type SQSTM1/p62 abrogated Ajuba-induced signalling significantly (p = 0.04). However, co-expression with UBA-deficient (ΔUBA) SQSTM1/p62 did not significantly decrease Ajuba activation of NF-κB.

### 3.2. UBA-deficient SQSTM1/p62 has increased interaction with Ajuba

**Fig 2:**
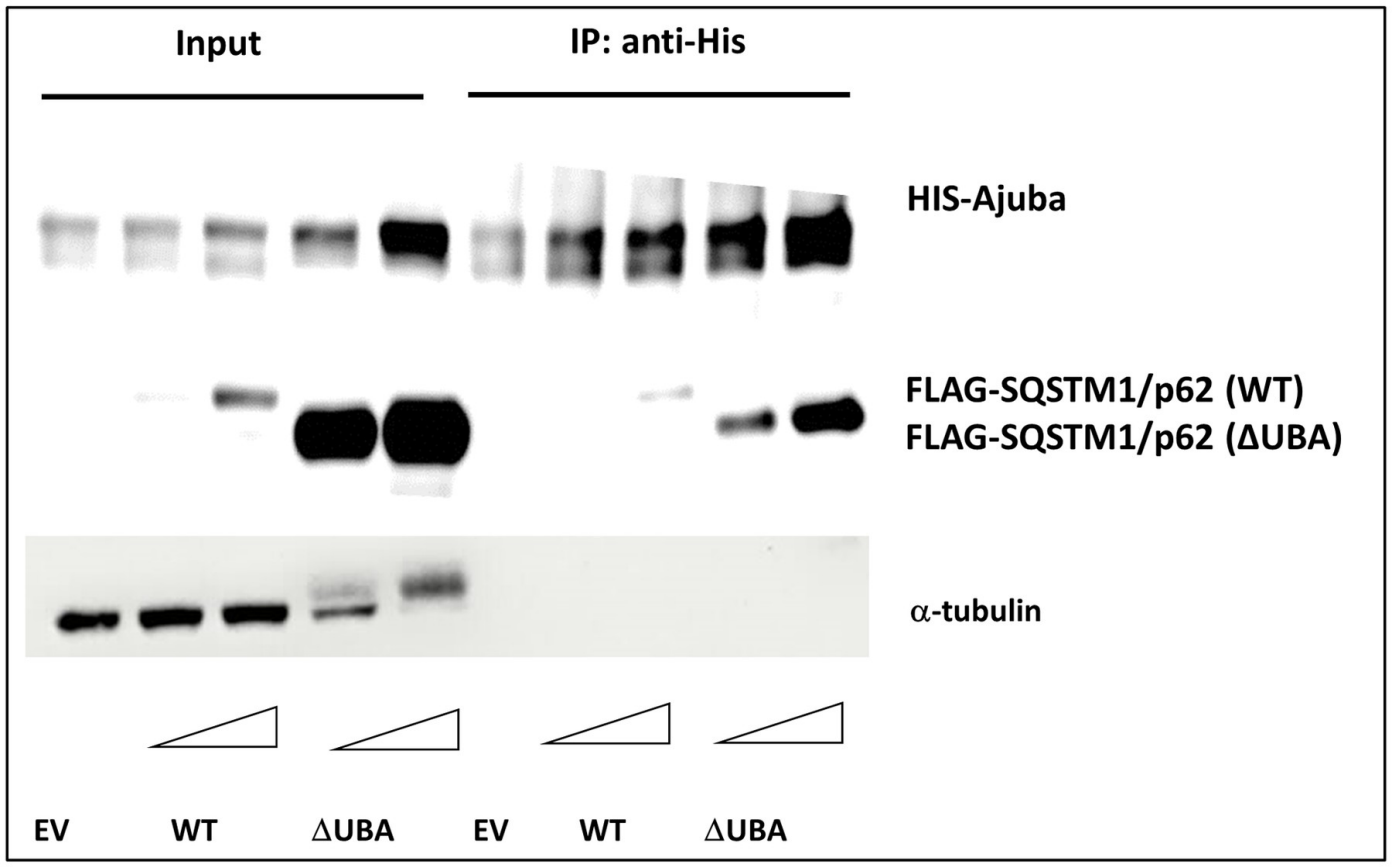
Ajuba interaction with UBA-deficient p62 is significantly greater than interaction with wild type. HEK293 cells were transfected with an expression plasmid for HIS-Ajuba, with either empty vector (EV) or increasing amounts of an expression plasmid for FLAG-p62 [WT or DUBA] as indicated. Forty-eight hours post-transfection cells were lysed and Ajuba immopurified with anti-HIS antibody. Ajuba and bound interacting proteins were separated through SDS polyacrylamide gel electrophoresis and transferred to membranes for Western blot analysis with anti-HIS (Ajuba) anti-FLAG (p62) antibodies. Images are representative of 3 independent experiments.

We reasoned that UBA-deficient SQSTM1/p62 may be unable to inhibit Ajuba activation of NF-κB due to altered signalling complex formation between Ajuba and SQSTM1/p62. Therefore, we investigated the interaction using co-immunoprecipitation experiments. Lysates were prepared from HEK293 cells co-transfected with empty vector and His_6_-Ajuba (control) or expression plasmids for FLAG-SQSTM1/p62 (wild type or ΔUBA) and His_6_-Ajuba. Pull-down of Ajuba with an anti-His antibody co-precipitated greater amounts of FLAG-SQSTM1/p62 ΔUBA than the wild type protein (Fig 2). Thus, SQSTM1/p62 ΔUBA interacts with Ajuba more readily than SQSTM1/p62 wild type.

### 3.3. Co-expression of SQSTM1/p62 has a protective effect on Ajuba levels

**Fig 3:**
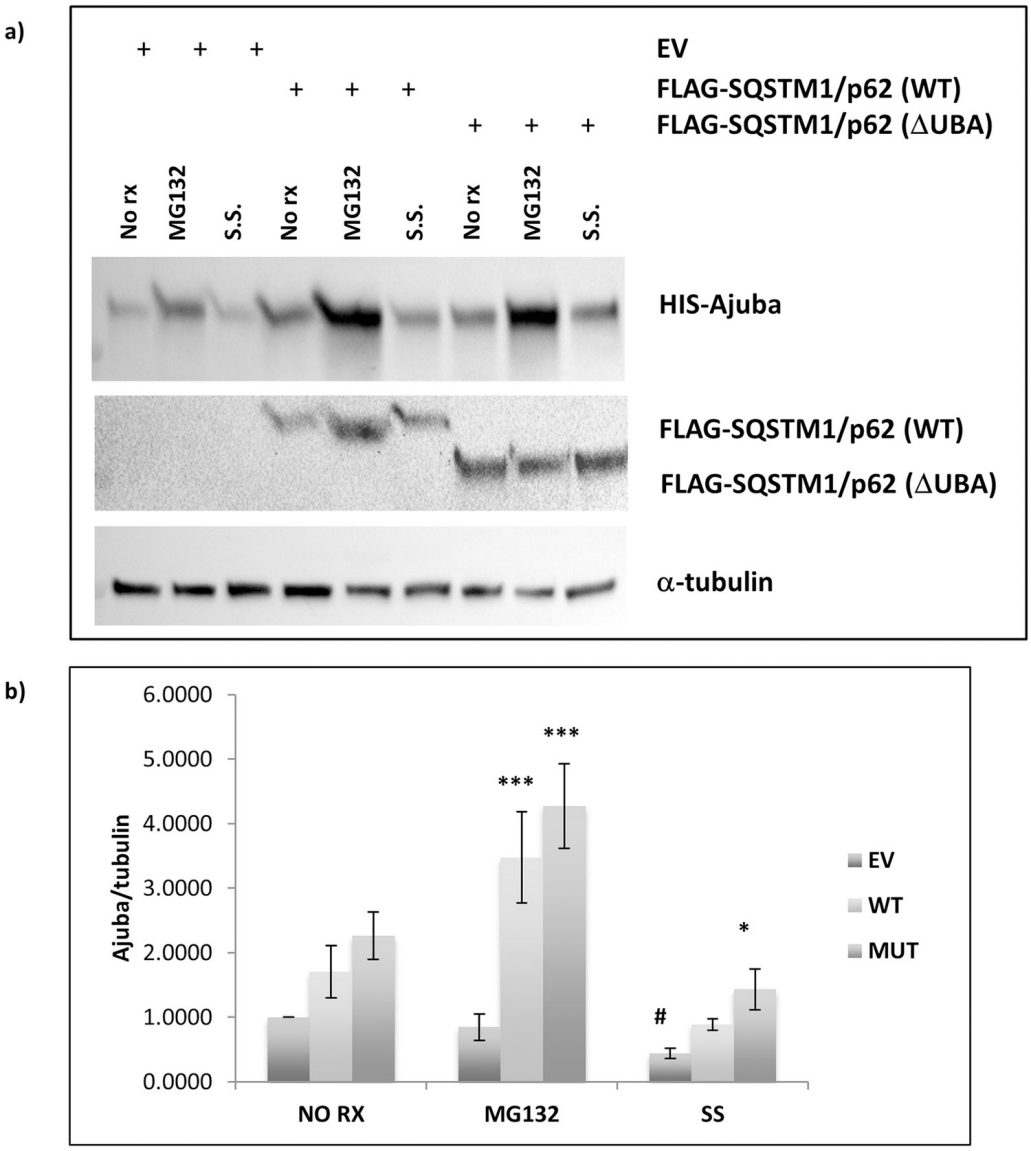
Proteasomal stress enhances the protective effect of p62 on Ajuba levels. HEK293 cells were transfected with pcDNA4/Xpress-HIS Ajuba and wild type p62 or ΔUBA p62 or empty vector (EV). Twenty-four hours post-transfection cells were (1) untreated or treated with (2) MG132, or (3) serum starved. Cells were lysed in RIPA buffer. A) Western blot analyses were performed using His (Ajuba), FLAG (SQSTM1/p62) and α-tubulin antibodies. Image is representative of 3 independent experiments. B) Densitometric analysis of Ajuba/α-tubulin. Data presented is the average of 3 independent experiments ± SEM. Compared to EV within treatment group, * p < 0.04 *** p < 0.01. Compared to EV non-treated, # p < 0.04.

We wondered whether expression of SQSTM1/p62 could be affecting the turnover of Ajuba. The UBA domain of SQSTM1/p62 is involved in protein degradation via both the ubiquitin-proteasome system and autophagy, therefore we treated cells expressing either SQSTM1/p62 wild type or ΔUBA overnight with either MG132, a proteasome inhibitor, or with serum starvation to induce autophagy, or left the cells untreated. We consistently observed that Ajuba levels were increased with expression of either wild type or ΔUBA SQSTM1/p62 proteins compared with cells transfected with empty vector control (Fig 3). In cells that were treated with MG132, co-expression with ΔUBA SQSTM1/p62 was associated with significantly greater detection of Ajuba (p < 0.001) compared with empty vector transfected MG132 treated cells. Similarly, co-expression with SQSTM1/p62 wild type led to higher levels of Ajuba in cells treated with MG132 (p < 0.01). Ajuba levels were significantly decreased in empty vector transfected cells that were serum starved compared with non-treated cells (p = 0.034) indicating that Ajuba was degraded by autophagy. However, there was no significant change in Ajuba levels in cells expressing SQSTM1/p62 (wild type or ΔUBA) that were serum starved compared with non-treated SQSTM1/p62 expressing cells (Fig 3).

### 3.4. Expression of SQSTM1/p62 affects Ajuba cellular localization

**Fig 4:**
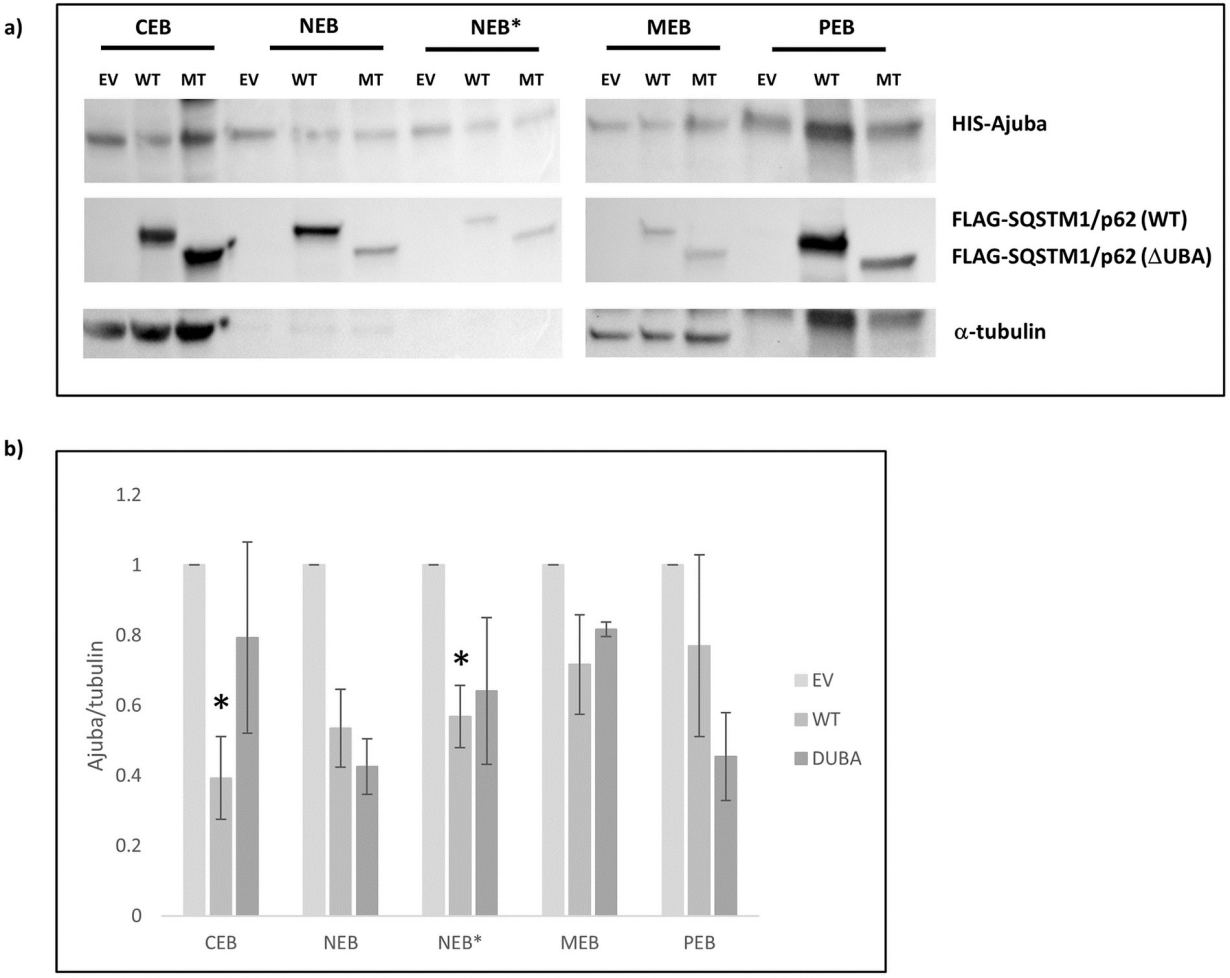
Increased expression of wild type p62 leads to a shift of Ajuba from the chromatin-bound fraction to the cytoskeleton. HEK293 cells were co-transfected with expression plasmids for Ajuba and SQSTM1/p62 (wild type [WT] or DUBA mutant [MT]) or empty vector (EV) as indicated. 48 hours post-transfection, cells were processed using a sub-cellular fractionation kit. a) Equal volumes of each fraction (CEB: Cytoplasmic extraction buffer, MEB: Membrane extraction buffer, NEB: Nuclear extraction buffer, NEB* Nuclear extraction buffer, chromatin-bound, PEB: Pellet extraction buffer) were separated through SDS PAGE and transferred to nitrocellulose membranes. Western blot analysis was performed with anti-Ajuba, anti-FLAG (SQSTM1/p62) and anti α-tubulin antibodies. Image is representative of n = 3 independent experiments. b) densitometric analysis of Ajuba/α-tubulin. The mean densitometries of each group form 4 independent experiments were compared with EV within each subcellular fraction. *p <0.04.

We hypothesised that SQSTM1/p62 inhibition of Ajuba cell signalling could be partially explained by altered subcellular localisation. We performed fractionation studies and found that expression with SQSTM1/p62 wild type (p = 0.009) or ΔUBA (p = 0.017) caused a significant mislocalisation of Ajuba away from the nuclear fraction (NEB) when compared with control cells (Fig 4). There were no significant alterations to Ajuba in other fractions.

## 4.0. Discussion

**Fig 5:**
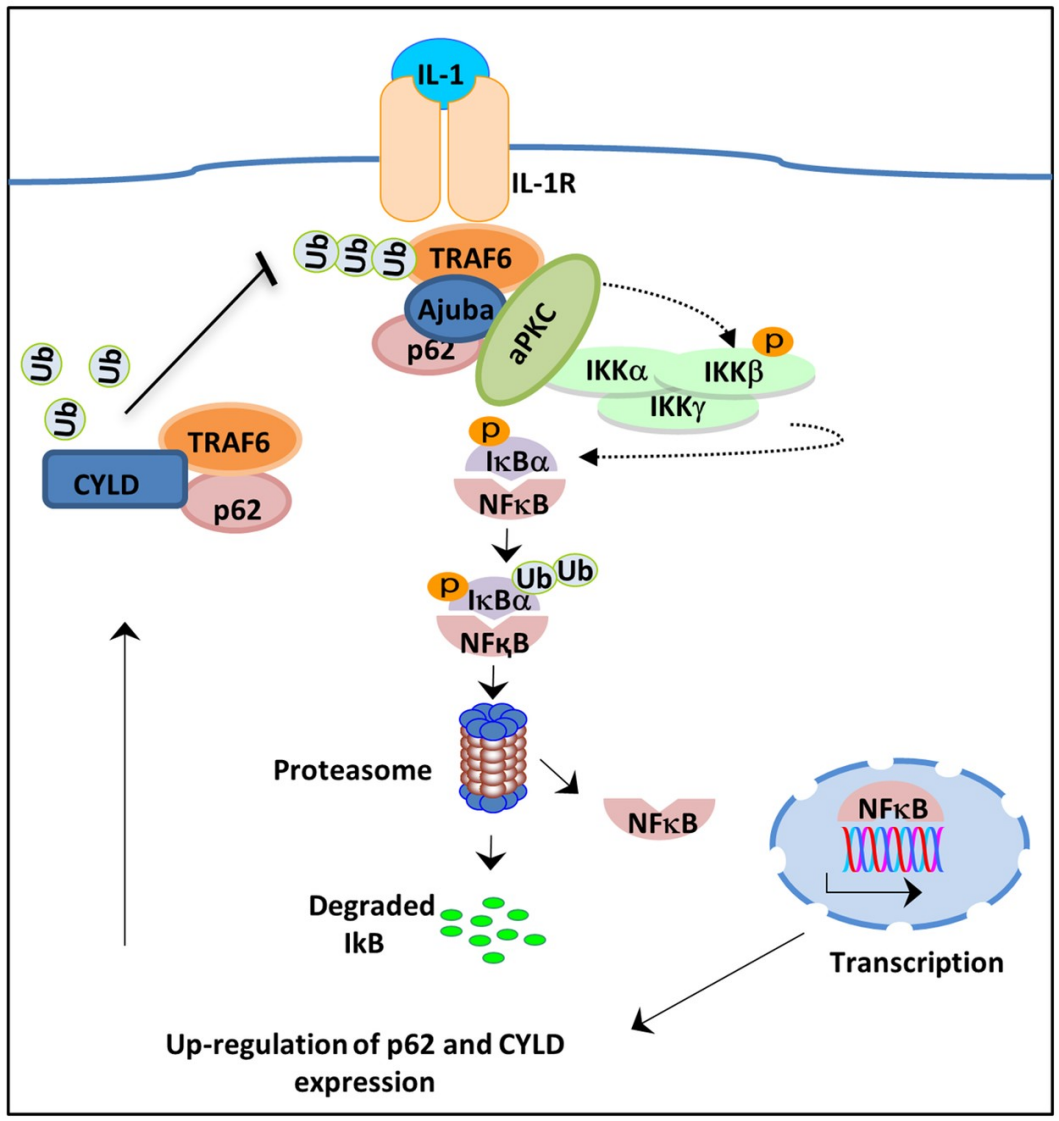
Interleukin-1 activation of NF-kB through Ajuba. Upon ligand binding TRAF6 binds to the Interleukin-1 receptor. Ajuba recruits p62 andFIG atypical protein kinase C (aPKC). aPKC phosphorylates the inhibitory kB kinase B, leading to its degradation and the release of NF-kB to affect gene transcription. NF-kB induces expression of p62 and the de-ubiquitination enzyme CYLD. This provides negative feedback to the pathway by de-ubiquitinating TRAF6, a process that is dependent on the UBA domain of p62. As such, the expression of p62 UBA domain mutant proteins does not provide negative feedback to the pathway and NF-kB remains activated.

Recently, the LIM domain protein Ajuba was reported to be an additional component of the SQSTM1/p62, TRAF6, aPKC complex required for efficient IL-1 signalling to NF-κB. SQSTM1/p62 is also an important regulator of RANKL-induced NF-κB in osteoclasts [6,16]. SQSTM1/p62 binds the adaptor protein TRAF6 and links it with aPKC, a kinase that phosphorylates IKKβ and thereby initiates downstream activation of NF-κB. However, SQSTM1/p62 is also a negative regulator of NF-κB signalling seemingly through the formation of TRAF6 complexes with the deubiquitinating enzyme CYLD that facilitates TRAF6 de-ubiquitination and thereby reduces NF-κB (Fig 5).

*SQSTM1* mutations affecting the UBA domain of SQSTM1/p62 are frequently reported in patients with Paget’s disease of bone and we have shown that SQSTM1/p62 mutant proteins are not able to inhibit NF-κB to the same extent as wild type SQSTM1/p62. Here we investigated whether SQSTM1/p62 regulates Ajuba-induced NF-κB signalling. We found that expressing Ajuba increased NF-κB activity compared with empty vector transfected control cells. Co-expression with wild type SQSTM1/p62 inhibited this function of Ajuba, whereas NF-κB signalling was unaffected in cells expressing ΔUBA SQSTM1/p62. Thus, inhibition of Ajuba-induced NF-κB by SQSTM1/p62 requires the UBA domain. This is in agreement with previous studies showing that Paget’s disease of bone-associated SQSTM1/p62 mutant proteins lose NF-κB repressive ability and may increase osteoclastogenesis due to an inability to provide negative feedback following RANKL-induced NF-κB activation. SQSTM1/p62 expression has previously been shown to inhibit LIMD1 AP-1 signalling. Thus, SQSTM1/p62 expression can re-direct signalling pathways such that they are not conducive to osteoclastogenesis. A limitation or study is that our results are in HEK293 cells as have previous studies [4,11–13], however similar results showing inhibition of NF-κB by SQSTM1/p62 and loss of this inhibition with a ΔUBA mutant have been shown in the osteoclast precursor RAW^264.7^ cells [14] indicating that with respect to NF-κB signalling the two cell types behave similarly.

We investigated whether an altered interaction between Ajuba and UBA-deficient SQSTM1/p62 might explain the lack of NF-κB repression we observed with the mutant SQSTM1/p62 protein. Using co-immunoprecipitations we observed that Ajuba pulled out more UBA-deficient SQSTM1/p62 than wild type SQSTM1/p62. It is possible that the negative regulation of NF-κB in the presence of Ajuba occurs via similar mechanisms shown in other studies in the absence of Ajuba and is mediated by UBA-domain dependent SQSTM1/p62 scaffolding of CYLD to TRAF6. Thus, ΔUBA SQSTM1/p62 appears to form signalling complexes with Ajuba that positively regulate NF-κB but does not facilitate the formation of negative signalling complexes that occur with wild type SQSTM1/p62.

We noted during our experiments that the amount of Ajuba observed in cells co-expressing SQSTM1/p62 was increased compared to empty-vector transfected cells. SQSTM1/p62 is a mediator of protein degradation, transporting ubiquitinated cargo proteins via the UBA domain to either the proteasome or the autophagosome. We investigated whether Ajuba may be a substrate of these pathways. We observed that Ajuba levels were significantly decreased in empty vector transfected cells following serum starvation, indicating that Ajuba is a substrate of autophagy. However, in cells expressing either SQSTM1/p62 wild type or ΔUBA, Ajuba did not decrease following serum starvation, indicating that SQSTM1/p62 co-expression has a protective effect against the autophagic degradation of Ajuba. Although we observed an increase in Ajuba levels in empty vector control cells treated with MG132, this did not reach significance. However, we observed that Ajuba levels were markedly increased in MG132 cells co-expressing SQSTM1/p62, both wild type and ΔUBA, and SQSTM1/p62 expression protects Ajuba levels in stressed cells in an UBA-independent manner. Together, our results show that Ajuba is a substrate of both the ubiquitin-proteasome system and the autophagy-lysosomal pathway.

We suggest that SQSTM1/p62 expression could be sequestering Ajuba into complexes that are protective against degradation. This sequestration may impact Ajuba functions. Ajuba has been implicated in a variety of cancers via its regulatory roles in numerous signalling pathways important for metastasis [17–20]. Over-expression of Ajuba suppressed the expression of Wnt target genes, important in cancer and development [21]. Ajuba is also a negative regulator of the Hippo pathway, implicated in control of tissue size and carcinogenesis and a role has been suggested for the pathway in osteoclastogenesis [17,22]. Ajuba has roles in cell migration as Ajuba^-/-^ MEFs show impaired migration [19]. Ajuba is also an important cytoskeletal protein and binds to the small GTPase Rac1, in both its inactive and active states, and stabilizes Rac1 at adherens junctions [23]. These studies implicate Ajuba as an important regulator of epithelial dynamics, which has implications for cancer and cell motility. The direct transcriptional effects of Ajuba on various signalling pathways may be impeded by SQSTM1/p62-mediated mislocalisation away from the nucleus, although Ajuba-mediated NF-κB is mediated by complex formation that lead to activation outside of the nucleus. Our results show that SQSTM1/p62 diverts both the cellular localisation and the NF-κB activating function of Ajuba. Additionally, we show for the first time that Ajuba is a substrate of autophagy and the proteasome and that SQSTM1/p62 protects Ajuba from degradation. However, these affects are independent.

## 5.0. Conclusions

Our results indicate that high levels of SQSTM1/p62 may act to sequester Ajuba into a holding compartment, that is not destined for signalling or degradation, and this is particularly evident during proteasomal stress. These findings may have implications for osteoclastogenesis in SQSTM1/p62-associated Paget’s disease of bone where mutant SQSTM1/p62 proteins are unable to repress Ajuba-mediated NF-κB signalling thus leading to increased osteoclastogenesis and may have effects on Ajuba-mediated Hippo signalling in osteoclasts. It is unknown whether Ajuba levels are increased in Paget’s disease of bone. Additionally our results may have implications for cancer states, where increased SQSTM1/p62 [24] and Ajuba [25] levels have been observed and have many key roles in cellular fate.

## Supporting information

S1 Raw images (PDF)

S1 Raw data (XLSX)
