# Supplementary material for: The SQSTM1/p62 UBA domain regulates Ajuba localisation, degradation and NF-κB signalling function
Source: PLoS One. 2021 Nov 4;16(11):e0259556. doi: 10.1371/journal.pone.0259556 (PMC8568271; doi:10.1371/journal.pone.0259556)
Supplement: S1 Raw images — (PDF) [file pone.0259556.s001.pdf]

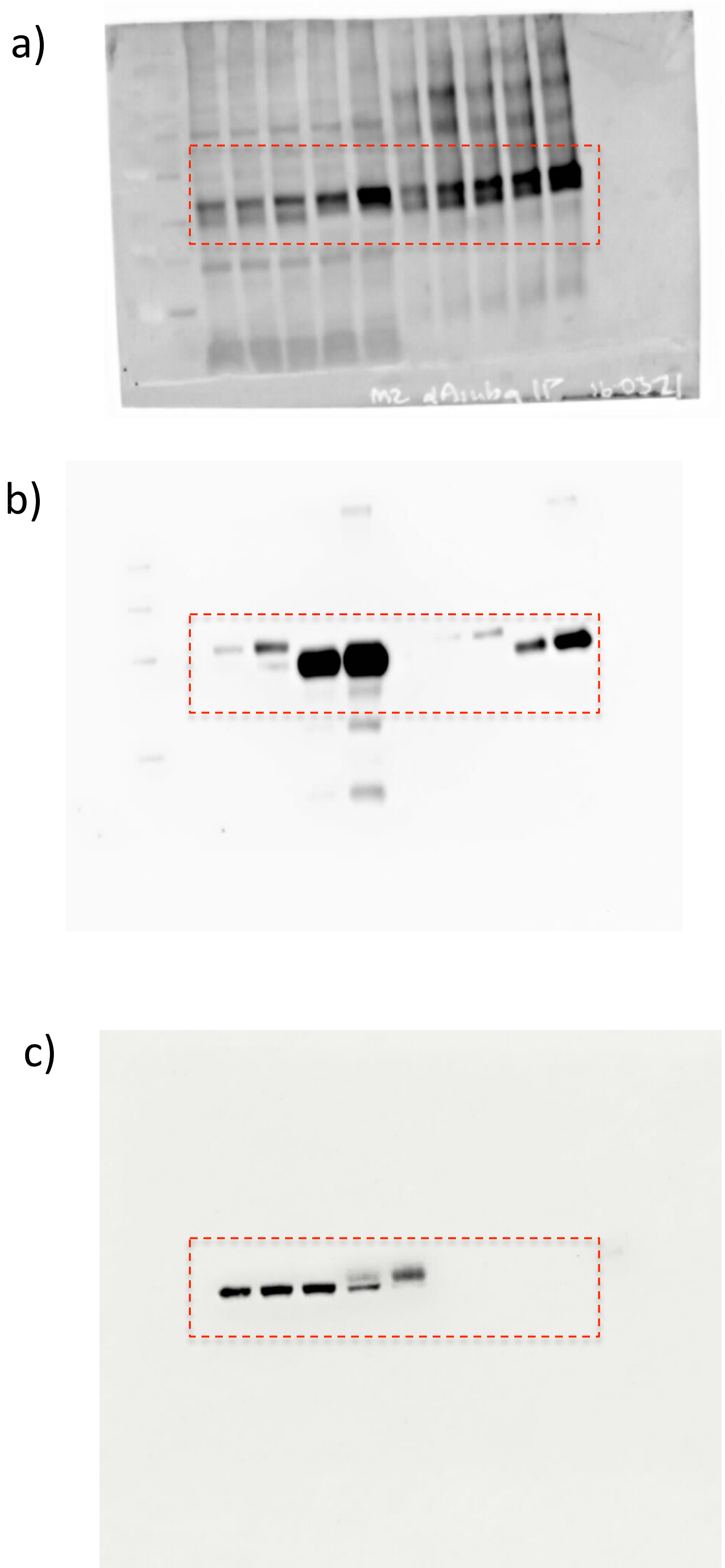

Fig S1. Ajuba interaction with UBA-deficient p62 is significantly greater than interaction with wild type – blot images. a) anti-Ajuba, b) anti-FLAG (p62) and c) anti-α-tubulin. Lane order: molecular weight marker, loading controls: empty vector, 1 μg FLAG-p62 WT, 4 μg FLAG-p62 WT, 1 μg FLAG-p62 ΔUBA, 4 μg FLAG-p62 ΔUBA, IP eluates: empty vector, 1 μg FLAG-p62 WT, 4 μg FLAG-p62 WT, 1 μg FLAG-p62 ΔUBA, 4 μg FLAG-p62 ΔUBA.

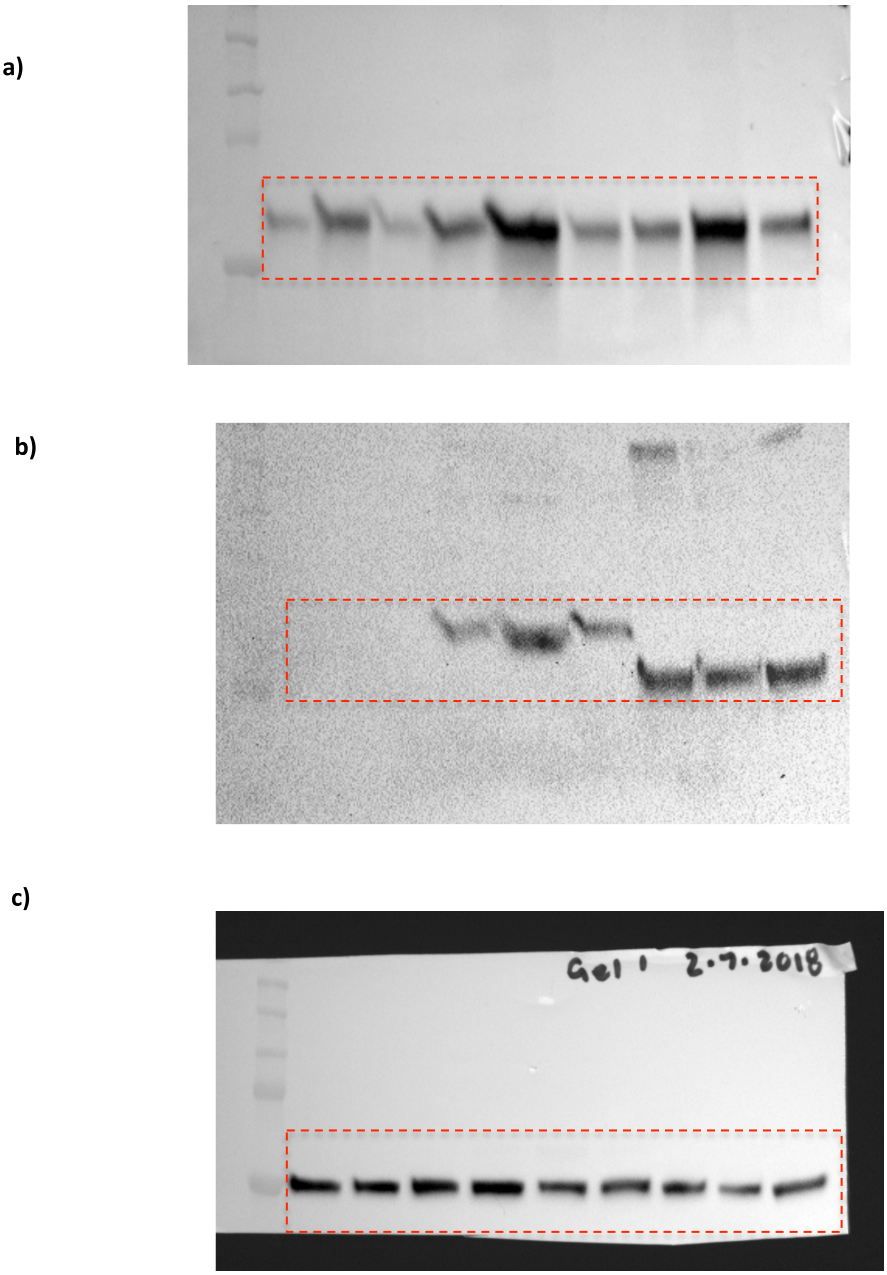

Fig S2. Proteasomal stress enhances the protective effect of p62 on Ajuba levels – blot images. A) anti-His (Ajuba), b) anti-FLAG (p62) and anti-α-tubulin. Lane order: molecular weight marker, empty vector no-treatment, MG132, serum starvation plus bafilomycin, FLAG-p62 wild type no-treatment, MG132, serum starvation plus bafilomycin, FLAG-p62 ΔUBA no-treatment, MG132, serum starvation plus bafilomycin.

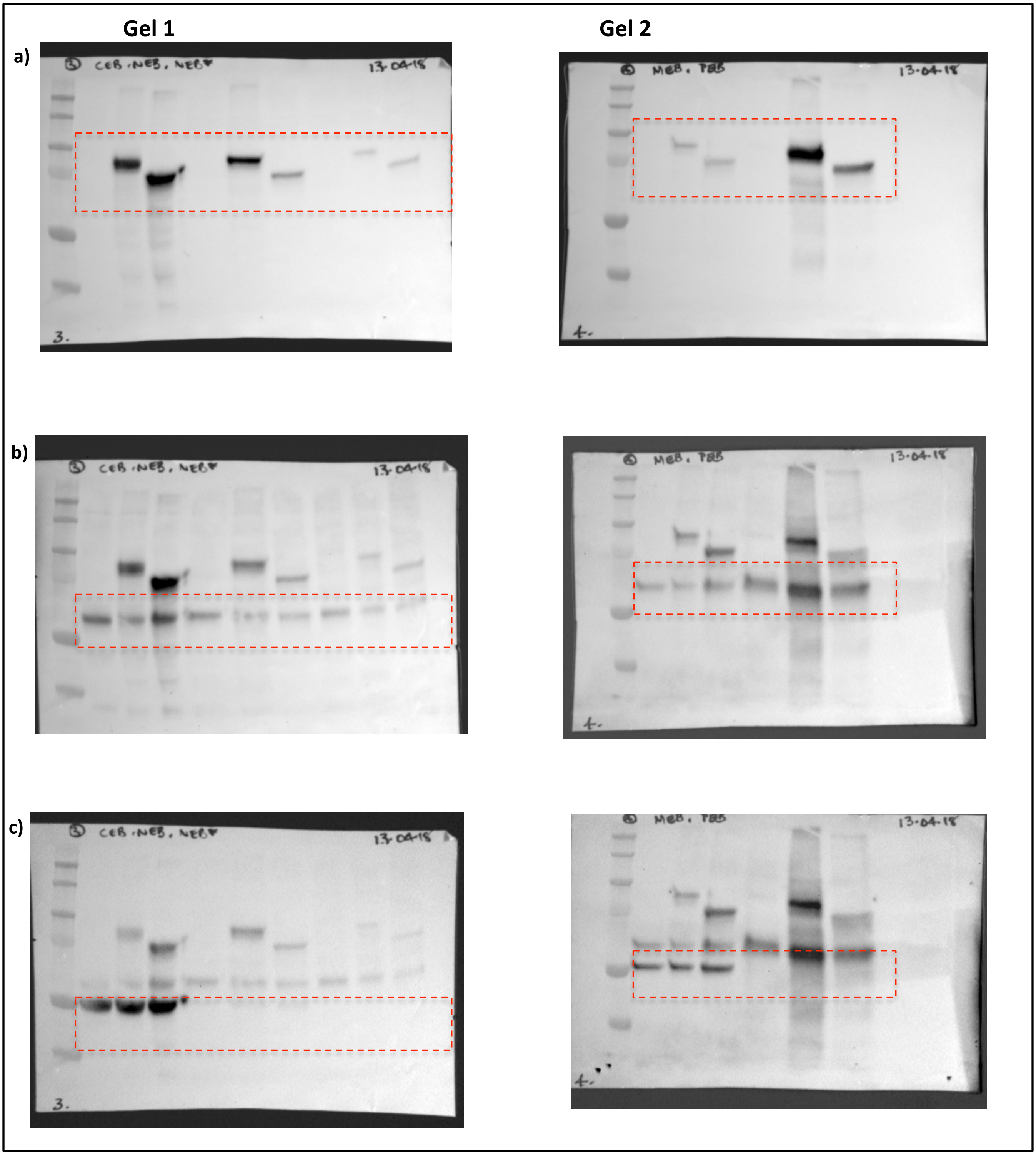

Fig S3. Increased expression of wild type p62 leads to a shift of Ajuba from the chromatin-bound fraction to the cytoskeleton − blot images. A) anti-FLAG (p62), b) anti-Ajuba [not stripped of anti-FLAG (p62)] and anti-α-tubulin (not stripped). Gel 1. Lane order: molecular weight marker, empty vector CEB, FLAG-p62 wild type CEB, FLAG-p62 ΔUBA CEB, empty vector NEB, FLAG-p62 wild type NEB, FLAG-p62 ΔUBA NEB, empty vector NEB*, FLAG-p62 wild type NEB*, FLAG-p62 ΔUBA NEB*. Gel 2. Lane order: molecular weight marker, empty vector MEB, FLAG-p62 wild type MEB, FLAG-p62 ΔUBA MEB, empty vector PEB, FLAG-p62 wild type PEB, FLAG-p62 ΔUBA PEB. CEB = cytoplasmic extraction buffer. NEB = nuclear extraction buffer. NEB* = nuclear extraction buffer, chromatin-bound . MEB = membrane extraction buffer . PEB = pellet extraction buffer (cytoskeletal) .
